# Supplementary material for: Global Phylogeography of Marine Synechococcus in Coastal Areas Reveals Strong Community Shifts
Source: mSystems. 2022 Dec 5;7(6):e00656-22. doi: 10.1128/msystems.00656-22 (PMC9765549; doi:10.1128/msystems.00656-22)
Supplement: FIG S1 [file msystems.00656-22-s0003.pdf]

Supplementary Figure

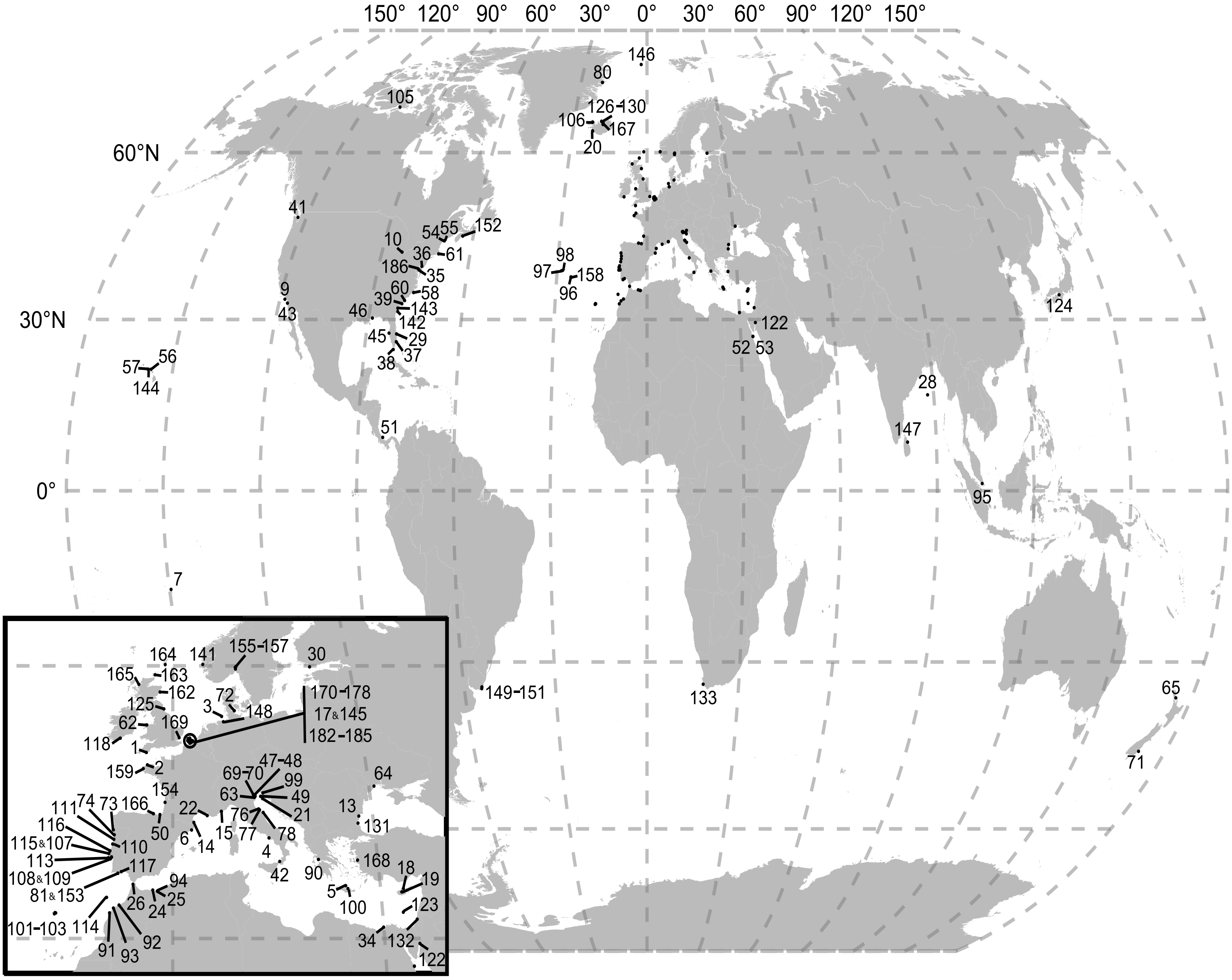

Supplementary Figure S1: **Map of OSD stations.**
All OSD stations that were analyzed in this study are indicated by their number.
The inset shows a close-up view of Europe.
