# Supplementary material for: Global Phylogeography of Marine Synechococcus in Coastal Areas Reveals Strong Community Shifts
Source: mSystems. 2022 Dec 5;7(6):e00656-22. doi: 10.1128/msystems.00656-22 (PMC9765549; doi:10.1128/msystems.00656-22)
Supplement: FIG S3 [file msystems.00656-22-s0005.pdf]

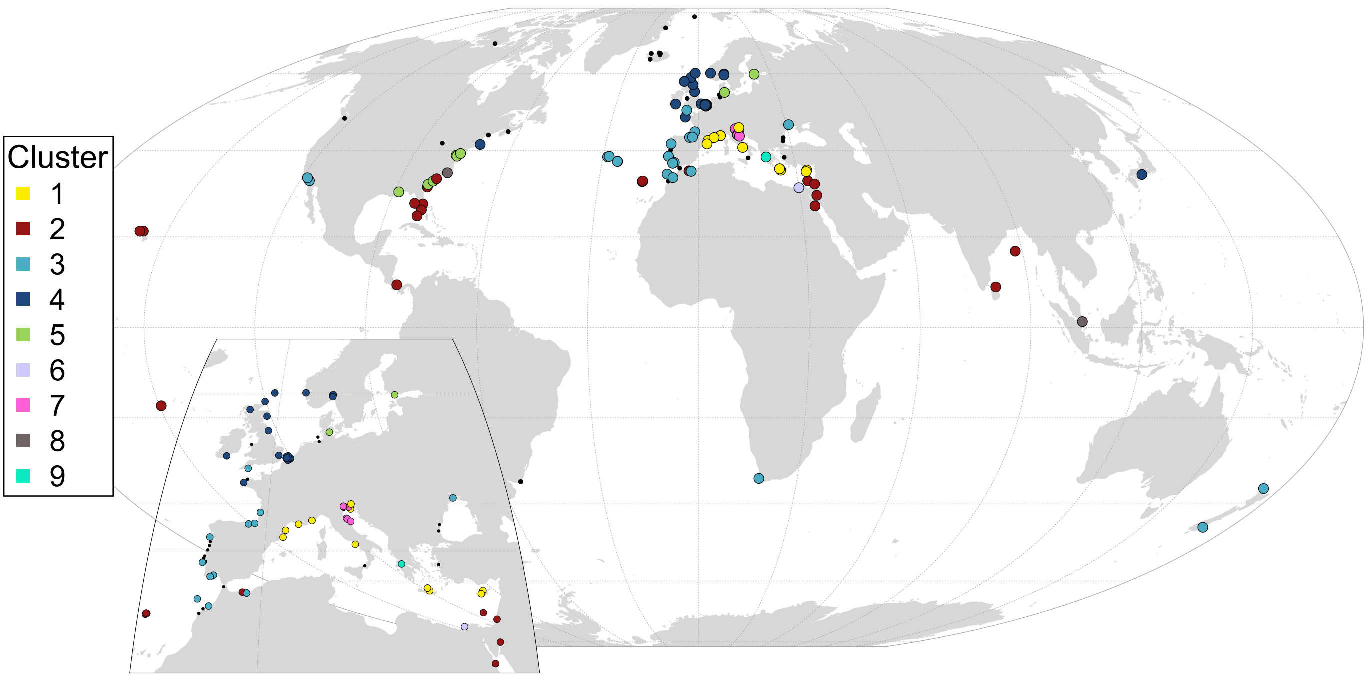

Figure S3: Global distribution of the clusters based on the relative abundance profiles of *Synechococcus* clades. The map shows Ocean Sampling Day stations (see Supplementary Figure S1), colored according to their cluster membership (see Figure 3 and Supplementary Figure S2).
