# Supplementary material for: Global Phylogeography of Marine Synechococcus in Coastal Areas Reveals Strong Community Shifts
Source: mSystems. 2022 Dec 5;7(6):e00656-22. doi: 10.1128/msystems.00656-22 (PMC9765549; doi:10.1128/msystems.00656-22)
Supplement: FIG S2 [file msystems.00656-22-s0004.pdf]

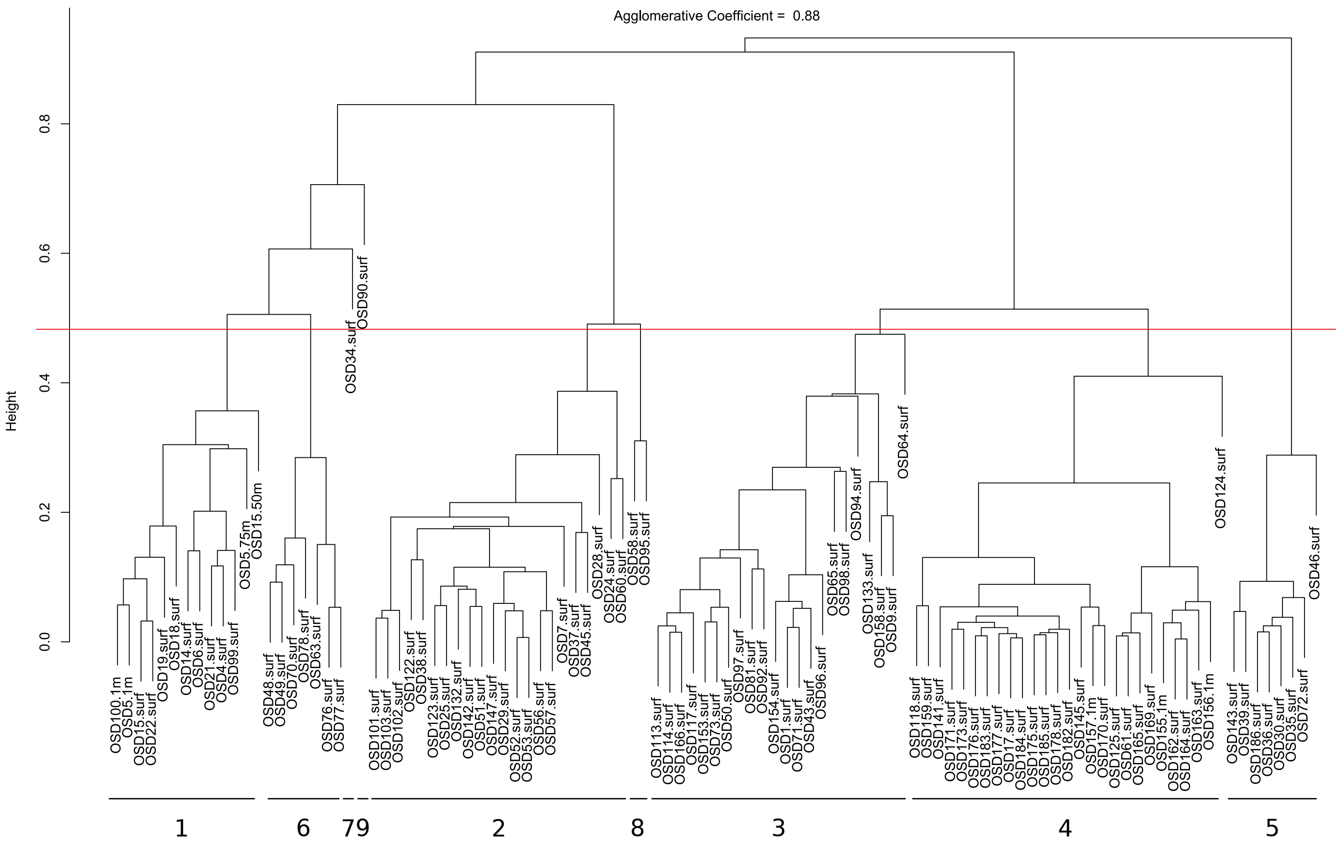

Figure S2: Clustering dendrogram Ocean Sampling Day (OSD) stations based on *Synechococcus* relative abundance profiles. OSD stations were clustered based on the relative abundance profiles of marine *Synechococcus* clades using Bray-Curtis distance: two stations will cluster together if they have a similar composition in *Synechococcus* clades. The red line indicates the level at which the dendrogram was cut to delineate the 9 clusters displayed on Figure 3.
